# Assessment of noninvasive brain stimulation interventions in Parkinson’s disease: a systematic review and network meta-analysis

**DOI:** 10.1038/s41598-024-64196-0

**Published:** 2024-06-20

**Authors:** Yueying Wang, Yi Ding, Chenchen Guo

**Affiliations:** 1 College of Rehabilitation Medicine, Shandong University of Traditional Chinese Medicine, Jinan, China; 2 Department of Rehabilitation Medicine, The Second Affiliated Hospital of Shandong University of Traditional Chinese Medicine, Jinan, China; 3 Department of Rehabilitation Medicine, Neck, Shoulder, Lumbago and Leg Pain Hospital Affiliated to Shandong First Medical University, Jinan, China

**Keywords:** Noninvasive brain stimulation, Parkinson’s disease, Systematic review, Network meta-analysis, Neuroscience, Diseases of the nervous system, Parkinson's disease

## Abstract

A network meta-analysis of randomized controlled trials was conducted to compare and rank the effectiveness of various noninvasive brain stimulation (NIBS) for Parkinson's disease (PD). We searched PubMed, Web of Science, Cochrane Library, Embase, China National Knowledge Infrastructure (CNKI), Wanfang Database, China Science and Technology Journal Database (VIP), and Chinese Biomedical Literature Service System (SinoMed) databases from the date of database inception to April 30th, 2024. Two researchers independently screened studies of NIBS treatment in patients with PD based on inclusion and exclusion criteria. Two researchers independently performed data extraction of the included studies using an Excel spreadsheet and assessed the quality of the literature according to the Cochrane Risk of Bias Assessment Tool (RoB2). Network meta-analysis was performed in StataMP 17.0. A total of 28 studies involving 1628 PD patients were included. The results showed that HF-rTMS over the SMA (SMD = − 2.01; 95% CI [− 2.87, − 1.15]), HF-rTMS over the M1 and DLPFC (SMD = − 1.80; 95% CI [− 2.90, − 0.70]), HF-rTMS over the M1 (SMD = − 1.10; 95% CI [− 1.55, − 0.65]), a-tDCS over the DLPFC (SMD = − 1.08; 95% CI [− 1.90, − 0.27]), HF-rTMS over the M1 and PFC (SMD = − 0.92; 95% CI [− 1.71, − 0.14]), LF-rTMS over the M1 (SMD = − 0.72; 95% CI [− 1.17, − 0.28]), and HF-rTMS over the DLPFC (SMD = − 0.70; 95% CI [− 1.21, − 0.19]) were significantly improved motor function compared with sham stimulation. The SUCRA three highest ranked were HF-rTMS over the SMA (95.1%), HF-rTMS over the M1 and DLPFC (89.6%), and HF-rTMS over the M1 (73.0%). In terms of enhanced cognitive function, HF-rTMS over the DLPFC (SMD = 0.80; 95% CI [0.03,1.56]) was significantly better than sham stimulation. The SUCRA three most highly ranked were a-tDCS over the M1 (69.8%), c-tDCS over the DLPFC (66.9%), and iTBS over the DLPFC (65.3%). HF-rTMS over the M1 (SMD = − 1.43; 95% CI [− 2.26, − 0.61]) and HF-rTMS over the DLPFC (SMD = − 0.79; 95% CI [− 1.45, − 0.12)]) significantly improved depression. The SUCRA three highest ranked were HF-rTMS over the M1 (94.1%), LF-rTMS over the M1 (71.8%), and HF-rTMS over the DLPFC (69.0%). HF-rTMS over the SMA may be the best option for improving motor symptoms in PD patients. a-tDCS and HF-rTMS over the M1 may be the NIBS with the most significant effects on cognition and depression, separately.

*Trial registration*: International Prospective Register of Systematic Review, PROSPERO (CRD42023456088)

## Introduction

Parkinson's disease (PD) is one of the most common complex neurodegenerative disorders in humans, caused mainly by degenerative necrosis of dopaminergic neurons in the dense portion of the substantia nigra, leading to decreased dopamine levels in the striatum^1–3^. In addition to motor symptoms such as bradykinesia and resting tremor, PD is associated with other non-motor symptoms, such as cognitive impairment and depression^4,5^. Dopaminergic drug replacement therapy, represented by levodopa, can alleviate most early PD symptoms^6^. However, it is essential to explore effective treatment methods actively because of the apparent adverse effects of drug therapy and the reduced efficacy of long-term use^7^.

Noninvasive brain stimulation (NIBS), safe and convenient neuromodulation techniques, have shown efficacy in improving movement, cognitive rehabilitation, and depression in PD and are considered to be more promising modalities of treatment^8–12^. The main types of NIBS used for PD include repetitive transcranial magnetic stimulation (rTMS), theta-burst stimulation (TBS), and transcranial direct current stimulation (tDCS). rTMS is a therapeutic technique that repeatedly stimulates the cerebral cortex by generating a magnetic field guided by a coil^13,14^. rTMS with a stimulation frequency > 1 Hz is called high-frequency rTMS (HF-rTMS), and rTMS with a stimulation frequency ≤ 1 Hz is called low-frequency rTMS (LF-rTMS)^15^. TBS is a specific mode of rTMS that enhances cortical excitability by mimicking cortical theta wave rhythms to enhance synaptic transmission and can be categorized into intermittent TBS (iTBS) and continuous TBS (cTBS) based on the time interval^16–18^. tDCS is a technique that applies low-intensity direct current to the scalp's surface to modulate cortical excitability^19^. An anodic electrode placed above the target area is called anodic tDCS (a-tDCS), while a cathodic electrode placed above the target area is called cathodic tDCS (c-tDCS). The stimulation targets of NIBS in PD patients mainly include the supplementary motor area (SMA), primary motor cortex (M1), dorsal lateral prefrontal cortex (DLPFC), and cerebellum^20–22^.

However, in most clinical studies using NIBS to improve PD symptoms, the sample sizes are small, and there is a wide variety of NIBS. To comprehensively compare the therapeutic effects of different NIBS, we performed a network meta-analysis to analyze the effects of NIBS on motor, cognitive, and depressive conditions in PD patients by evaluating multiple scales to inform clinical practice.

## Methods

### Protocol and registration

This study was conducted following the Preferred Reporting Items for Systematic Reviews and Meta-Analyses (PRISMA) statement 2020 guideline^23,24^ and A MeaSurement Tool to Assess Systematic Reviews (AMSTAR) 2^25^. The registration of this study was completed with the International Prospective Register of Systematic Review, PROSPERO (CRD42023456088).

### Search strategy

Computer searches of PubMed, Web of Science, Cochrane Library, Embase, China National Knowledge Infrastructure (CNKI), Wanfang Database, China Science and Technology Journal Database (VIP), and Chinese Biomedical Literature Service System (SinoMed) databases were performed from construction to April 30th, 2024. The search languages were English and Chinese. We searched ClinicalTrials.gov for gray literature and unpublished studies. In addition, we manually searched references for included studies, review articles and meta-analysis. The whole strategy, with search terms for each database, is accessible in Supplementary Table S1.

### Inclusion and exclusion criteria

The inclusion criteria included: (1) Patient: adults (≥ 18 years) with PD who meet the diagnostic criteria for PD, regardless of gender, race, or disease severity; (2) Intervention: NIBS stimulation, with an unlimited number of NIBS sessions, stimulation parameters, and target locations; (3) Comparator: sham NIBS; (4) Outcomes: indicators of motor function were the motor section of the Unified Parkinson's Disease Rating Scale (UPDRS-III) and the motor section of the Movement Disorder Society Unified Parkinson's Disease Rating Scale (MDS-UPDRS-III); indicators of cognitive function assessment in non-motor function were the Mini-Mental State Examination (MMSE) and the Montreal Cognitive Assessment (MoCA); indicators of depression assessment in non-motor function were the Beck Depression Inventory (BDI) and the Hamilton Depression Rating Scale (HDRS); (5) randomized controlled trials (RCTs).

The exclusion criteria included: (1) duplicate publications or duplicate literature data; (2) study data not available; (3) not RCT;(4) protocol but not report of study result.

### Study selection and data extraction

Two researchers independently screened titles and abstracts after removing duplicates and subsequently reviewed the full text based on predetermined criteria to identify eligible studies and perform data extraction. Any disagreements were resolved through discussion with the third researcher. The following information was independently extracted for the included studies using an Excel sheet: first author, time of publication, number of study participants, gender, age, course of disease and severity, intervention modality, NIBS parameters, site of stimulation, and treatment duration, follow-up time after treatment, outcome indicators and results after treatment, and state of medication.

### Risk of *bias* assessment

According to the Cochrane risk of bias tool (RoB2), two researchers individually assessed each of the five sections: randomization process, deviations from intended interventions, missing outcome data, measurement of outcome, and selection of reported result^26^. We determined the risk of bias to be low, some concerns, or high by using the RoB2 to answer important questions for each of these sections. If each section is low risk, the overall risk of bias is "low risk"; if more than one section is "some concerns" and there is no "high risk", the overall risk of bias is "some concerns"; as long as one section is "high risk", the overall risk of bias is "high risk". Inconsistent evaluations were discussed and finalized with the third researcher.

### Data synthesis and analysis

The outcome measures in this study were continuous variables, and the mean and standard deviation (SD) of the change in scores in each scale before and after treatment were calculated according to the formulas in the Cochrane Handbook for Systematic Reviews of Interventions to eliminate baseline differences^26^.

$$\begin{aligned} & Mean_{{{\text{change}}}} = Mean_{{{\text{final}}}} - Mean_{{{\text{baseline}}}} \\ & SD_{{{\text{change}}}} = \sqrt {SD_{{{\text{baseline}}}}^{2} + SD_{{{\text{final}}}}^{2} - (2 \times Corr \times SD_{{\text{baseline }}} \times SD_{{{\text{final}}}} )} \\ & {\text{Corr = 0}}{.5} \\ \end{aligned}$$

Network meta-analysis was performed in StataMP 17.0 using the "network meta" command. A network relationship plot was performed in which the circles indicate the sample size of included studies, and the straight lines indicate the number of studies between the two interventions. When a closed loop exists, direct and indirect comparison consistency was assessed using the node-splitting method, with *P* > 0.05 indicating good consistency, which can be analyzed using the consistency model, and vice versa using the inconsistency model. In addition, we evaluated the efficacy of different sham NIBS stimulations using pairwise meta-analysis with the Comprehensive Meta-Analysis software 3.7 to demonstrate the assumption of transitivity of network meta-analysis^27,28^. Forest plots of NIBS compared to sham stimulation were drawn. League tables for pairwise meta-analysis were made. The surface under the cumulative ranking curve (SUCRA) was calculated to perform the superiority ranking of the interventions. The closer the SUCRA value was to 100%, the higher the probability that the intervention would be optimal. Funnel plots were drawn for publication bias analysis.

We used the Grading of Recommendations Assessment, Development and Evaluation (GRADE) rating tool to assess the quality of the analyzed evidence^29^. We assessed quality by categorizing the outcome indicators into four levels high quality, moderate quality, low quality, and very low quality based on five dimensions: study limitations, imprecision, inconsistency, indirectness, and publication bias.

## Results

### Literature selection and characteristics of the included literatures

**Figure 1:**
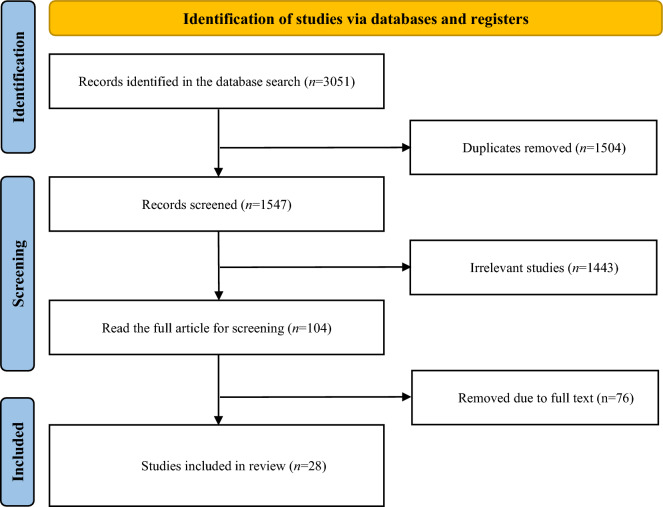
The flowchart of the literature screening process.

A total of 3051 articles were initially retrieved from the database. After removing 1504 duplicate articles, 1443 studies were excluded after initial screening. Of the remaining 104 articles, 76 were excluded after reviewing the full text based on inclusion and exclusion criteria. Finally, 28 studies were selected for network meta-analysis. A flowchart of the study screening process is shown in Fig. 1, and a list of excluded studies and the reasons for their exclusion are shown in Supplementary Table S2. NIBS methods for the included studies included rTMS^30–46^, iTBS^47,48^, and tDCS^49–57^. The studies included 1628 PD patients, the NIBS group with 966, and the sham NIBS group with 662. The sample sizes of the NIBS and sham NIBS groups ranged from 7–54 individuals. The characteristics of the included studies are shown in Table 1.

**Table 1:** The characteristics of the included studies.

References	Sample size (E/C)	Gender (male/female)	Age (E/C, year)	Course of disease (E/C, year/month)	H&Y Stage (1/2/3/4/5)	Intervention	Site of stimulation	Treatment duration	Follow-up	Outcome	State of medication (I/E)
Benninger et al.^49^	13/12	9/4;7/5	(63.6 ± 9.0)/(64.2 ± 8.8)	(10.6 ± 7.1)/(9.1 ± 3.3) y	2–4	2 mA tDCS	M1 + SMA	3 d/wk, 2.5 wks	1 mon; 3 mons	①	On/on & off
Benninger^47^	13/13	7/6;11/2	(62.1 ± 6.9)/(65.6 ± 9.0)	(10.8 ± 7.1)/(6.5 ± 3.4) y	2–4	iTBS, 80% RMT	M1 + DLPFC	4 d/wk, 2 wks	1 mon	①	On/on & off
Shirota et al.^30^	34/36/36	12/22;14/22; 19/17	(67.9 ± 8.4)/(68.8 ± 7.6)/(65.7 ± 8.5)	(7.8 ± 6.6)/(8.5 ± 7.3)/(7.6 ± 4.4) y	0/9/21/4/0; 0/10/21/5/0; 0/10/21/5/0	10 Hz rTMS, 110% RMT; 1 Hz rTMS, 110% RMT	SMA	8 wks	12 wks	⑤	On/on
Biundo et al.^50^	7/9	6/1;8/1	(69.1 ± 7.6)/(72.3 ± 4.1)	–	1–3	2 mA tDCS	DLPFC	20 min/d, 4 d/wk, 4 wks	16 wks	①④⑥	On/–
Li^31^	30/30/30	15/15;16/14; 16/14	(65.3 ± 8.1)/(66.1 ± 7.6)/(66.5 ± 7.5)	(6.6 ± 5.3)/(6.1 ± 5.2)/(6.4 ± 4.9) y	–	5 Hz rTMS, 90–100% RMT;0.5 Hz rTMS, 90–100% RMT	DLPFC	2 d/wk,4 wks	–	①⑤	On/–
Yu et al.^32^	31/33	14/17;16/17	(67.25 ± 6.71)/(68 ± 7.56)	(2.76 ± 1.56)/(2.64 ± 1.49) y	1–2	5 Hz rTMS	DLPFC	10 days	1 mon	①⑤	On/off
Li et al.^51^	28/28	14/14;15/13	(64.32 ± 5.59)/(64.39 ± 5.5)	(1.19 ± 0.57)/(1.28 ± 0.56) y	(1.3 ± 0.4)/(1.2 ± 0.5)	2 mA tDCS	M1	20 min/d, 8 wks	–	④	–
Khedr et al.^33^	26/26	40/22	(59.58 ± 11.28)/(55.88 ± 13.84)	(4.60 ± 3.64)/(4.85 ± 3.39) y	–	20 Hz rTMS, 90% RMT;1 Hz rTMS, 100% RMT	M1	10 days	1 mon	①	On/on
Yang et al.^34^	17/17	20/14	48–76	–	–	1 Hz rTMS, 80% RMT	M1	20 min/d, 20 days	–	①	On/–
Mi et al.^35^	20/10	9/11;5/5	(62.65 ± 10.56)/(65.60 ± 8.68)	(9.15 ± 5.82)/(7.40 ± 4.83) y	(2.60 ± 0.85)/(2.35 ± 0.91)	10 Hz rTMS, 90% RMT	SMA	5 d/wk, 2 wks	2wks;4 wks	②	On/on
Chung et al.^36^	17/17/16	10/7;9/8;7/9	(62.7 ± 6.8)/(62.1 ± 5.7)/(62.1 ± 5.7)	(5.2 ± 3.4)/(7.5 ± 4.9)/(6.9 ± 3.3) y	(2.2 ± 0.3)/(2.2 ± 0.4)/(2.3 ± 0.3)	25 Hz rTMS, 80% RMT;1 Hz rTMS, 80% RMT	M1	4 d/wk,3 wks	1 mon;3 mons	②	On/on
Guo^37^	38/38/38	18/20;17/21;19/19	(65.91 ± 3.42)/(66.28 ± 3.55)/(66.57 ± 3.39)	(6.48 ± 2.08)/(6.15 ± 1.97)/(6.64 ± 2.11) y	0/22/16/0/0;0/23/15/0/0;0/20/18/0/0	5 Hz rTMS, 100% RMT;1 Hz rTMS, 100% RMT	M1	10 days	1 mon	①③⑤	On/–
Lai et al.^38^	20/20	12/8;14/6	(69.55 ± 1.64)/(71.2 ± 1.67)	(4.23 ± 0.61)/(5.5 ± 1.28) y	–	10 Hz rTMS, 80% RMT	SMA	5 d/wk,4 wks	–	①	On/–
Spagnolo et al.^39^	19/20/20	12/7;15/5;14/6	(63.9 ± 10)/(60.4 ± 8.1)/(64.2 ± 5.5)	(7.6 ± 4.9)/(5.8 ± 2.1)/(7.2 ± 3) y	2(2–2.5);2(2,2);2(2,2)	10 Hz rTMS, M1: 90% RMT; PFC:100% RMT	M1 + PFC; M1	3 d/wk,4 wks	–	②③⑥	On/off
Sun et al.^52^	11/11	4/7;9/2	(62 ± 14.73)/(65 ± 12.67)	(8.2 ± 3.8)/(7.6 ± 3.2) y	1–3	2 mA tDCS	DLPFC	20 min/d,5 d/wk,4 wks	–	③④	On/–
Wu et al.^53^	28/26	16/12;14/12	(61 ± 11.6)/(62.6 ± 12.2)	(5.8 ± 2.6)/(5.7 ± 3.5) y	(2.4 ± 0.8)/(2.5 ± 0.6)	1.2 mA tDCS	DLPFC	20 min/d,5 d/wk,4 wks	–	⑤	On/on
Aftanas et al.^40^	23/23	12/11;9/14	(63.7 ± 8.8)/(62.9 ± 7.1)	(7.0 ± 4.0)/(5.6 ± 4.0) y	0/10/13/0/0;0/11/12/0/0	10 Hz rTMS, M1: 100% RMT; DLPEC:110% RMT	M1 + DLPFC	40 min/d,3 wks	–	②③⑤⑥	On/–
He et al.^48^	20/15	13/7;10/5	(70.0 ± 6.3)/(74.8 ± 6.9)	(2.7 ± 1.5)/(2.5 ± 1.1) y	(2.7 ± 1.1)/(2.5 ± 1.0)	iTBS, 100% RMT	DLPFC	5 d/wk,2 wks	3 mons	④	On/on
Hu et al.^54^	49/49	30/19;28/21	(64.23 ± 4.78)/(63.68 ± 5.22)	(33.02 ± 10.65)/(32.32 ± 12.44) mon	–	2 mA tDCS	DLPFC	45 min/d, 12 wks	–	③④	On/–
Lee and Kim^55^	15/15	6/9;8/7	(70.00 ± 3.76)/(71.33 ± 3.27)	(6.27 ± 1.03)/(7.00 ± 1.41) mon	(2.47 ± 0.52)/(2.80 ± 0.41)	2 mA tDCS	M1	20 min/d, 5 d/wk,4 wks	2 wks	①	On/–
Liao et al.^41^	30/30	17/13;16/14	(59.03 ± 6.84)/(60.43 ± 6.94)	(1.89 ± 0.63)/(1.92 ± 0.59) y	1–2.5	10 Hz rTMS, 90% RMT	DLPFC	25 min/d,5 d/wk,4 wks	–	④	–
Chen et al.^42^	32/32	19/13;18/14	(65.21 ± 5.32)/(65.32 ± 5.24)	(2.59 ± 0.61)/(2.65 ± 0.63) y	(2.16 ± 0.5)/(2.19 ± 0.52)	1 Hz rTMS	DLPFC	20 min/d,5 d/wk,4 wks	–	②	On/–
Dong et al.^43^	49/49	27/22;26/23	(66.02 ± 4.83)/(65.73 ± 4.97)	(5.81 ± 1.41)/(5.89 ± 1.35) y	2–3	10 Hz rTMS, 90% RMT;1 Hz rTMS, 90% RMT	M1	5 d/wk,4 wks	–	①⑤	On/–
Hong et al.^57^	30/30	17/13;18/12	(68.16 ± 3.97)/(68.34 ± 4.29)	(2.38 ± 0.72)/(2.34 ± 0.86) y	8/8/7/7/0;9/7/8/6/0	2 mA tDCS	DLPFC	20 min/d,5 d/wk,2 wks	–	①④	On/–
Wang et al.^56^	43/42	26/17;23/19	(64.41 ± 5.65)/(63.96 ± 6.49)	–	–	2 mA tDCS	DLPFC	25 min/d,5 d/wk,3 mons	–	③④	On/–
Zheng et al.^45^	54/54	26/28;24/30	(66.83 ± 7.86)/(67.70 ± 8.41)	–	1–3	5 Hz rTMS, 110% RMT	DLPFC	5 d/wk,4 wks	1 mon	①③⑤	On/on
Zhou et al.^44^	12/12	5/7;6/6	(70.75 ± 7.83)/(70.42 ± 8.99)	5(3.25, 9.75);5.5(2.5, 9.75) y	1/7/2/2/0;2/5/3/2/0	1 Hz rTMS, 120% RMT	M1	1d/wk, 4wks	–	①	On/on
Wang et al.^46^	41/41	30/11;32/9	(60.52 ± 2.35)/(60.15 ± 2.32)	–	–	25 Hz rTMS, 90% RMT	DLPFC	5 d/wk,4 wks	3 mons	④⑤	–

Data presented as mean ± SD or median (interquartile range, IQR); E/C, E, experiment group/control group; I/E, intervention/evaluation; PFC, prefrontal cortex; RMT, resting motor threshold; ① UPDRS-III; ② MDS-UPDRS-III; ③MMSE; ④ MoCA; ⑤ HDRS; ⑥ BDI-II

### Risk of *bias* of included literatures

**Figure 2:**
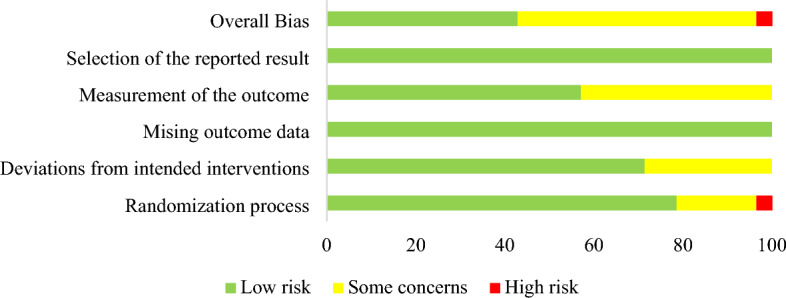
Risk of bias summary.

42.9% of studies^33,35,36,40,45,47–50,53^ showed a low overall risk of bias. 53.6%^30–32,34,37–39,42–44,46,51,52,54–57^ of studies expressed some concerns about the risk of bias. 3.6% of studies^41^ showed a high overall risk of bias. The risk of bias was mainly due to unclear randomization methods or allocation processes^32,39,41,42,51,54^, inability to ensure blinding of intervention implementers due to research needs^30,31,34,37,42,46,52,56^, and uncertainty as to whether the study blinded the outcome assessors^31,32,34,37,38,41–44,46,55,57^. A summary of the risk of bias is shown in Fig. 2.

### Assessment of motor function improvement

**Figure 3:**
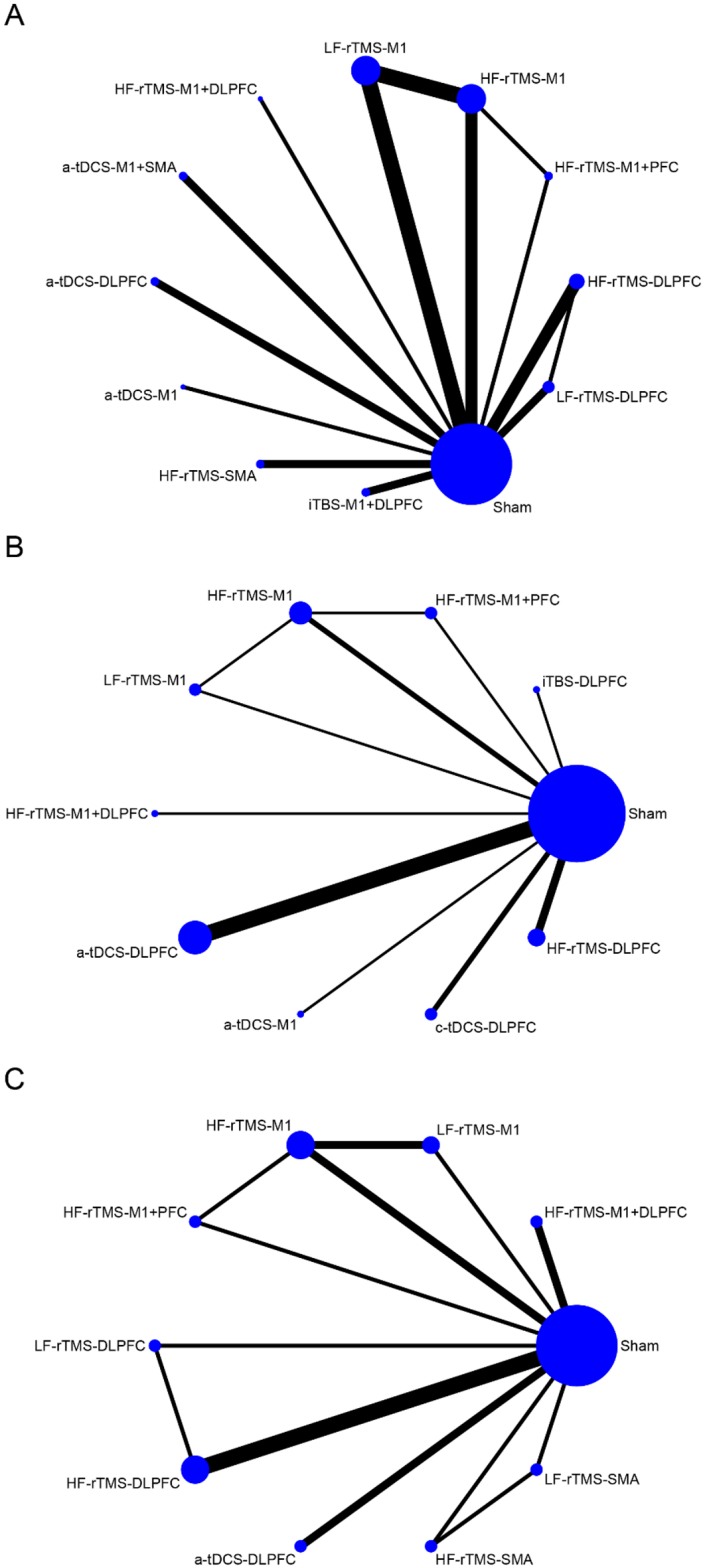
Network relationship plots. (**A**) motor function (**B**) cognitive function (**C**) depression.

**Figure 4:**
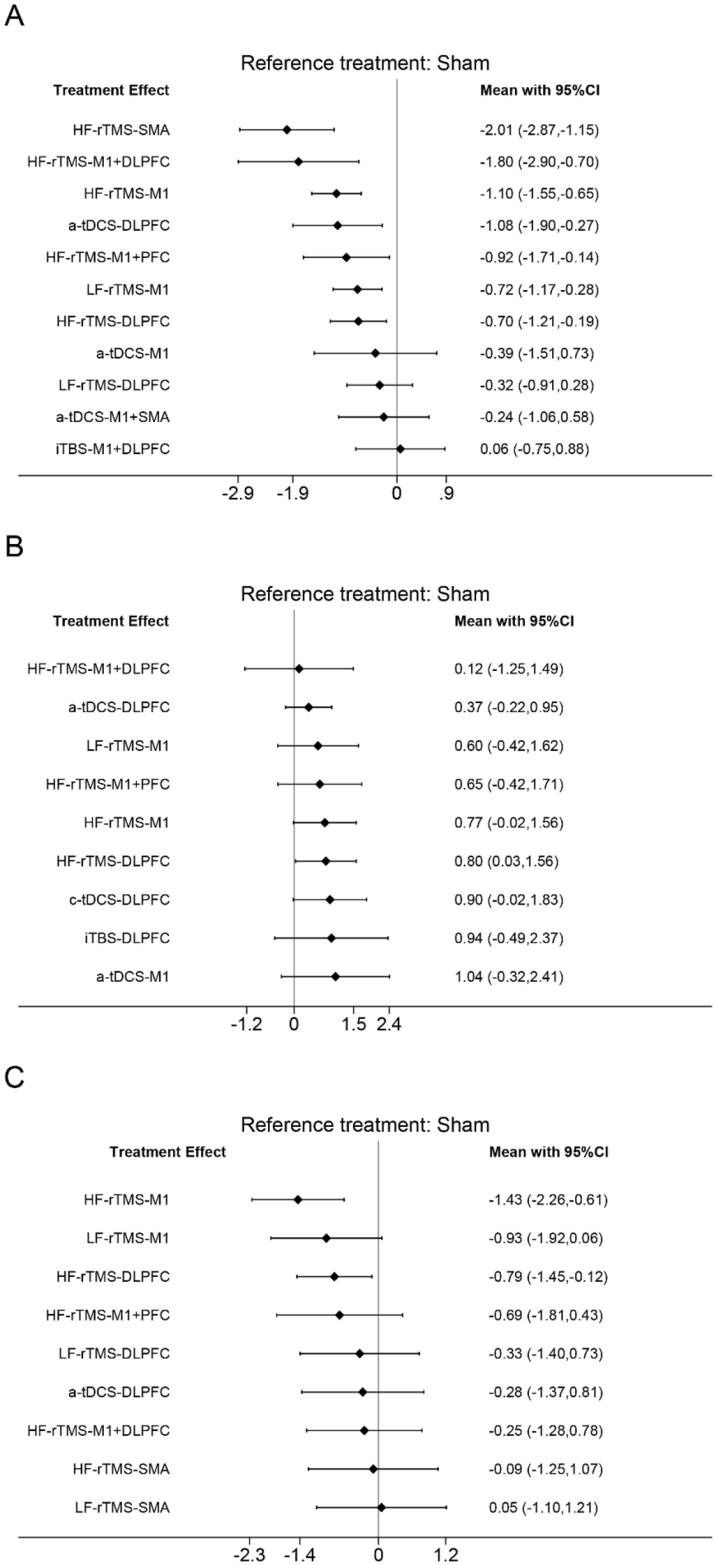
Forest plots for direct comparison with sham stimulation. (**A**) motor function (**B**) cognitive function (**C**) depression.

**Figure 5:**
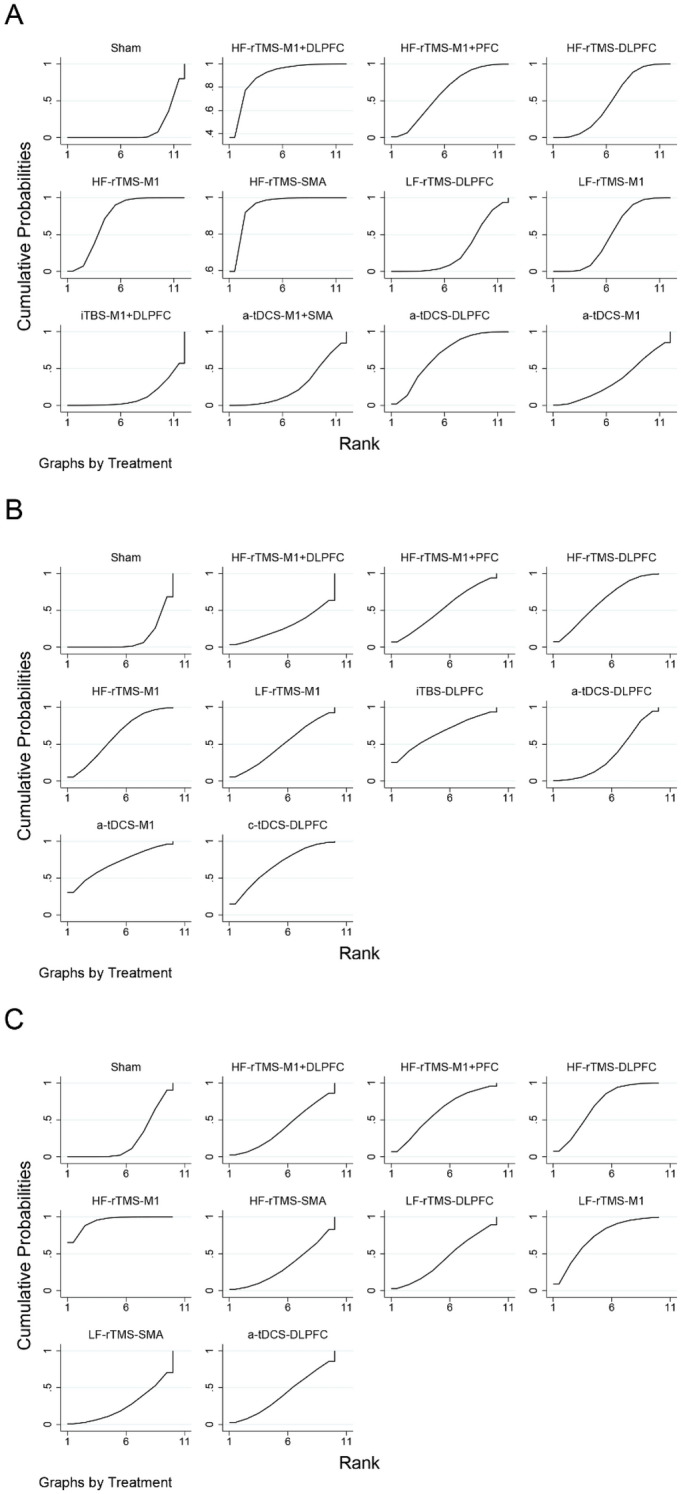
Probability rankings based on SUCRA. (**A**) motor function (**B**) cognitive function (**C**) depression.

As shown in Fig. 3A, the network meta-analysis reporting motor function in patients with PD contains 12 interventions that form 14 pairs of direct comparisons. The node-splitting method reports that this closed-loop local inconsistency is not significant (Supplementary Table S3). The sham NIBS treatment effect was not statistically different between sham iTBS, sham rTMS, and sham tDCS treatments (*P* = 0.378) (Supplementary Figure 1). The pairwise meta-analysis of NIBS compared with sham stimulation showed that HF-rTMS over the SMA (SMD = − 2.01; 95% CI [− 2.87, − 1.15]), HF-rTMS over the M1 and DLPFC (SMD = − 1.80; 95% CI [− 2.90, − 0.70]), HF-rTMS over the M1 (SMD = − 1.10; 95% CI [− 1.55, − 0.65]), a-tDCS over the DLPFC (SMD = − 1.08; 95% CI [− 1.90, − 0.27]), HF-rTMS over the M1 and PFC (SMD = − 0.92; 95% CI [− 1.71, − 0.14]), LF-rTMS over the M1 (SMD = − 0.72; 95% CI [− 1.17, − 0.28]), and HF-rTMS over the DLPFC (SMD = − 0.70; 95% CI [− 1.21, − 0.19]) significantly improved motor function (Fig. 4A, Table 2). According to SUCRA, HF-rTMS over the SMA (95.1%) ranked the highest probability of being the best therapy, followed by HF-rTMS over the M1 and DLPFC (89.6%) and HF-rTMS over the M1 (73.0%) (Fig. 5A, Table 3).

**Table 2:**
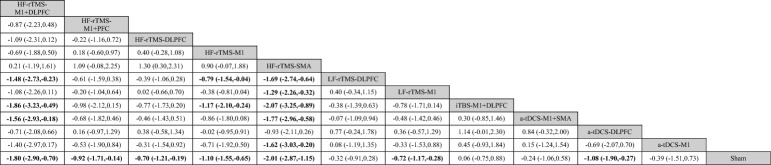
League table of the changes of motor function.

Bold results marked with indicate statistical significance.

**Table 3:** SUCRA of the changes of motor function.

Treatment	SUCRA (%)
HF-rTMS-SMA	95.1
HF-rTMS-M1 + DLPFC	89.6
HF-rTMS-M1	73.0
a-tDCS-DLPFC	67.7
HF-rTMS-M1 + PFC	61.1
HF-rTMS-DLPFC	50.4
LF-rTMS-M1	50.0
a-tDCS-M1	34.5
LF-rTMS-DLPFC	28.3
a-tDCS-M1 + SMA	26.3
iTBS-M1 + DLPFC	12.6
Sham	11.3

### Assessment of cognitive function improvement

As shown in Fig. 3B, the network meta-analysis reporting cognitive functioning in patients with PD contains 10 interventions that form 11 pairs of direct comparisons. The node-splitting method shows no significant local inconsistency in this network plot (Supplementary Table S4). The difference in the efficacy of sham NIBS treatment was not significant between sham iTBS, sham rTMS, and sham tDCS treatments (*P* = 0.055) (Supplementary Figure 2). However, the efficacy was significant in the sham tDCS group (SMD = 1.052; 95% CI [0.599, 1.504]). The pairwise meta-analysis with sham stimulation showed that HF-rTMS over the DLPFC (SMD = 0.80; 95% CI [0.03,1.56]) significantly enhanced cognitive function (Fig. 4B, Table 4). The probability of a-tDCS over the M1 (69.8%) being the optimal therapy is the highest according to SUCRA, followed by c-tDCS over the DLPFC (66.9%) and iTBS over the DLPFC (65.3%) (Fig. 5B, Table 5).

**Table 4:**
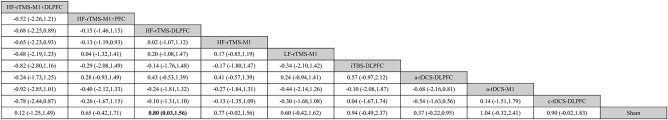
League table of the changes of cognitive function.

Bold result marked with indicate statistical significance.

**Table 5:** SUCRA of the changes of cognitive function.

Treatment	SUCRA (%)
a-tDCS-M1	69.8
c-tDCS-DLPFC	66.9
iTBS-DLPFC	65.3
HF-rTMS-DLPFC	61.5
HF-rTMS-M1	60.8
HF-rTMS-M1 + PFC	52.1
LF-rTMS-M1	48.7
a-tDCS-DLPFC	35.5
HF-rTMS-M1 + DLPFC	27.9
Sham	11.3

### Assessment of depression improvement

As shown in Fig. 3C, the network meta-analysis reporting depression in patients with PD contained 10 interventions that formed 13 pairwise direct comparisons. The node-splitting method shows that local inconsistency is insignificant in this closed loop (Supplementary Table S5). The sham NIBS treatment effect was not significantly different between sham rTMS and sham tDCS treatments (*P* = 0.875) (Supplementary Figure 3). The NIBS and sham stimulation pairwise meta-analysis showed that HF-rTMS over the M1 (SMD = − 1.43; 95% CI [− 2.26, − 0.61]) and HF-rTMS over the DLPFC (SMD = − 0.79; 95% CI [− 1.45, − 0.12)]) significantly improved depression (Fig. 4C, Table 6). Based on SUCRA, HF-rTMS over the M1 (94.1%) has the highest probability of being the optimal treatment followed by LF-rTMS over the M1 (71.8%) and HF-rTMS over the DLPFC (69.0%) (Fig. 5C, Table 7).

**Table 6:**
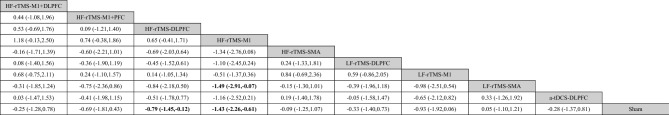
League table of the changes of depression.

Bold results marked with indicate statistical significance.

**Table 7:** SUCRA of the changes of depression.

Treatment	SUCRA (%)
HF-rTMS-M1	94.1
LF-rTMS-M1	71.8
HF-rTMS-DLPFC	69.0
HF-rTMS-M1 + PFC	60.7
LF-rTMS-DLPFC	43.1
a-tDCS-DLPFC	40.6
HF-rTMS-M1 + DLPFC	39.5
HF-rTMS-SMA	33.2
LF-rTMS-SMA	25.6
Sham	22.5

### Publication *bias*

**Figure 6:**
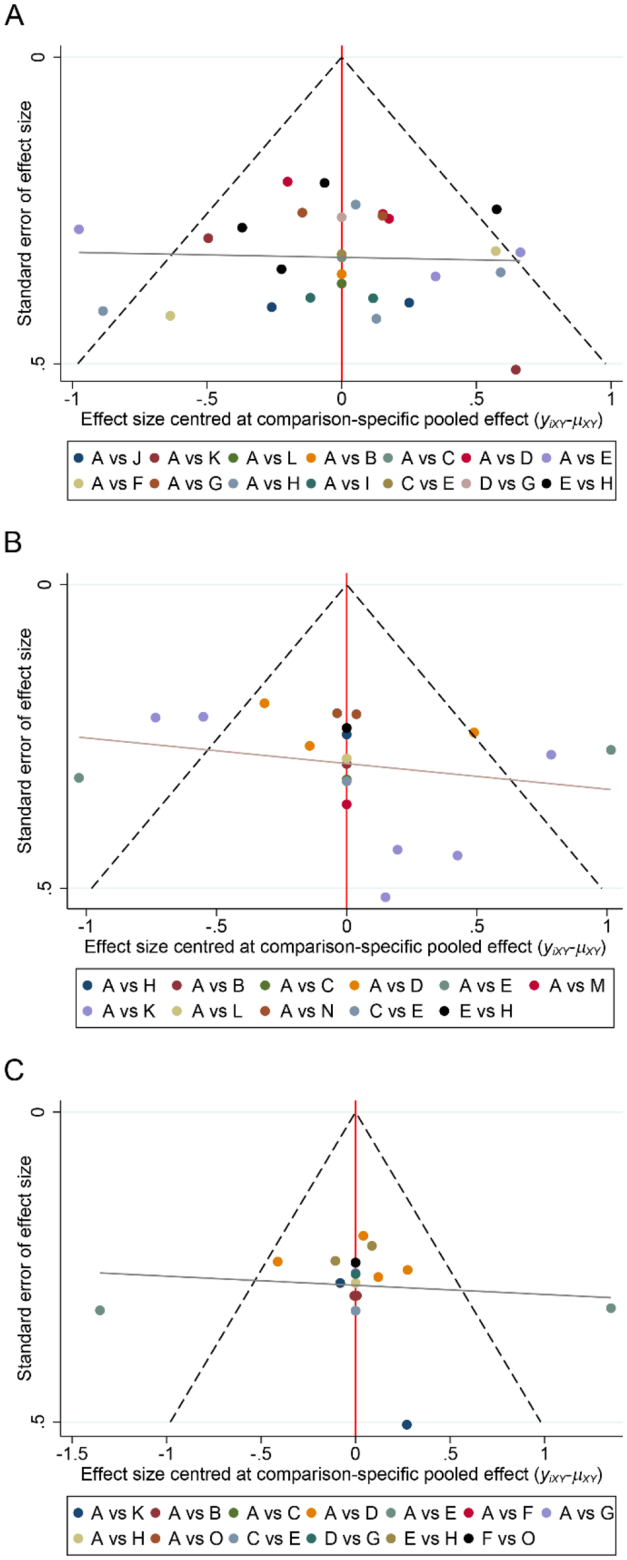
Funnel plots. (**A**) motor function (**B**) cognitive function (**C**) depression. A, Sham; B, HF-rTMS-M1 + DLPFC; C, HF-rTMS-M1 + PFC; D, HF-rTMS-DLPFC; E, HF-rTMS-M1; F, HF-rTMS-SMA; G, LF-rTMS-DLPFC; H, LF-rTMS-M1; I, iTBS-M1 + DLPFC; J, a-tDCS-M1 + SMA; K, a-tDCS-DLPFC; L, a-tDCS-M1; M, iTBS-DLPFC; N, c-tDCS-DLPFC; O, LF-rTMS-SMA.

Funnel plots using motor function, cognitive function, and depression status as outcome indicators were all generally symmetrical, suggesting no significant publication bias (Fig. 6A–C).

### GRADE ratings

The results of the GRADE evaluation are shown in Table 8. In summary, the overall quality of the overall evidence was low to moderate. It was mainly due to some risk of bias in the included studies, 95% confidence intervals crossing the clinical decision threshold, and some heterogeneity among the combined studies, which affected the scientific validity of the research methodology and the reliability of the findings.

**Table 8:** GRADE evaluation quality of evidence.

Comparisons	Study limitations	Imprecision	Inconsistency	Indirectness	Publication bias	GRADE
HF-rTMS-M1 + DLPFC versus Sham	No downgrade	Downgraded because 95% CI passes through the equivalence line	No downgrade	No downgrade	No downgrade	⊕⊕⊕◯ Moderate
HF-rTMS-M1 + PFC versus Sham	Downgraded because moderate RoB2 comparisons > 70%	Downgraded because 95% CI passes through the equivalence line	No downgrade	No downgrade	No downgrade	⊕⊕◯◯ Low
HF-rTMS-M1 + PFC versus HF-rTMS-M1	No downgrade	Downgraded because 95% CI passes through the equivalence line	No downgrade	No downgrade	No downgrade	⊕⊕⊕◯ Moderate
HF-rTMS-DLPFC versus Sham	No downgrade	No downgrade	Downgraded because *I*^2^ > 50%	No downgrade	No downgrade	⊕⊕⊕◯ Moderate
HF-rTMS-DLPFC versus LF-rTMS-DLPFC	Downgraded because moderate RoB2 comparisons > 70%	Downgraded because 95% CI passes through the equivalence line	No downgrade	No downgrade	No downgrade	⊕⊕◯◯ Low
HF-rTMS-M1 versus Sham	No downgrade	No downgrade	Downgraded because* I*^2^ > 50%	No downgrade	Downgraded because of incomplete symmetry of scatter points in the funnel plot	⊕⊕◯◯ Low
HF-rTMS-M1 versus LF-rTMS-M1	No downgrade	Downgraded because 95% CI passes through the equivalence line	Downgraded because* I*^2^ > 50%	No downgrade	Downgraded because of incomplete symmetry of scatter points in the funnel plot	⊕◯◯◯ Very Low
HF-rTMS-SMA versus Sham	No downgrade	Downgraded because 95% CI passes through the equivalence line	Downgraded because* I*^2^ > 50%	No downgrade	No downgrade	⊕⊕◯◯ Low
HF-rTMS-SMA versus LF-rTMS-SMA	Downgraded because moderate RoB2 comparisons > 70%	Downgraded because 95% CI passes through the equivalence line	No downgrade	No downgrade	No downgrade	⊕⊕◯◯ Low
LF-rTMS-DLPFC versus Sham	Downgraded because moderate RoB2 comparisons > 70%	Downgraded because 95% CI passes through the equivalence line	No downgrade	No downgrade	No downgrade	⊕⊕◯◯ Low
LF-rTMS-M1 versus Sham	Downgraded because moderate RoB2 comparisons > 70%	No downgrade	Downgraded because* I*^2^ > 50%	No downgrade	No downgrade	⊕⊕◯◯ Low
iTBS-M1 + DLPFC versus Sham	No downgrade	Downgraded because 95% CI passes through the equivalence line	No downgrade	No downgrade	No downgrade	⊕⊕⊕◯ Moderate
a-tDCS-M1 + SMA versus Sham	No downgrade	Downgraded because 95% CI passes through the equivalence line	No downgrade	No downgrade	No downgrade	⊕⊕⊕◯ Moderate
a-tDCS-DLPFC versus Sham	Downgraded because moderate RoB2 comparisons > 70%	No downgrade	Downgraded because* I*^2^ > 50%	No downgrade	No downgrade	⊕⊕◯◯ Low
a-tDCS-M1 versus Sham	Downgraded because moderate RoB2 comparisons > 70%	Downgraded because 95% CI passes through the equivalence line	No downgrade	No downgrade	No downgrade	⊕⊕◯◯ Low
iTBS-DLPFC versus Sham	No downgrade	Downgraded because 95% CI passes through the equivalence line	No downgrade	No downgrade	No downgrade	⊕⊕⊕◯ Moderate
c-tDCS-DLPFC versus Sham	Downgraded because moderate RoB2 comparisons > 70%	Downgraded because 95% CI passes through the equivalence line	No downgrade	No downgrade	No downgrade	⊕⊕◯◯ Low
LF-rTMS-SMA versus Sham	No downgrade	Downgraded because 95% CI passes through the equivalence line	No downgrade	No downgrade	No downgrade	⊕⊕⊕◯ Moderate

We grade based on the following criteria estimates.

(1) Study limitations: We downgraded by one level when the contributions from low RoB2 comparisons were less than 30% and contributions from moderate RoB2 comparisons were 70% or greater.

(2) Imprecision: We determined whether the confidence intervals crossed the clinical decision thresholds for recommended and non-recommended treatments. If it crossed it was downgraded for imprecision.

(3) Inconsistency: We based our ratings on heterogeneity tests and inconsistency tests. Downgrade if there is significant heterogeneity (*I*^2^ > 50%) or inconsistency (*P* < 0.05).

(4) Indirectness: We analyzed the efficacy of different sham NIBS by pairwise meta-analysis methods to ensure network transitivity. The results of our analysis proved the transitivity (*P* > 0.05).

(5) Publication bias: We assessed this based on the symmetry of the comparison-correction funnel plot and the funding sources and stakes of the included study.

## Discussion

This study is based on 28 RCTs using network meta-analysis to assess the efficacy of different NIBS in the treatment of PD and to help in choosing the best option for clinical treatment. We found that most NIBS protocols improved motor function in patients with PD. Specifically, HF-rTMS over the SMA was found to be most effectively associated with improved motor function. In terms of cognitive function, SUCRA results showed that a-tDCS over the M1 was considered most effectively associated with its improvement. Notably, the results of pairwise meta-analysis showed that only HF-rTMS over the DLPFC was significantly more efficacious than the sham stimulation group in the different NIBS. HF-rTMS over the M1 was found to be most effectively associated with improved depression.

A primary finding of the study results was that HF-rTMS was effective in improving motor dysfunction in patients with PD, which is consistent with the conclusions of a previous network meta-analysis^58^. We further comparatively investigated the target areas of action of rTMS and found that SMA may be more effective in the treatment of motor disorders. SMA is a key brain region that connects the motor and cognitive nervous systems and plays an important role in motor preparation and control^59^. SMA dysfunction is considered to be an important cause of continuous motor abnormalities and gait disturbances in PD patients. Resting-state functional magnetic resonance imaging study showed significant differences in functional connectivity in sensorimotor, insula, and cerebellum networks between PD patients and healthy individuals^60^.

The second primary finding of the study results is that a-tDCS over the M1 and HF-rTMS over the M1 may be better for cognition and depression separately. However, there was no statistically significant difference in efficacy between a-tDCS over the M1 compared to the sham stimulation group. Therefore, these findings should be interpreted cautiously to ensure that future large-scale randomized controlled trials provide additional evidence. Patients with PD suffer from dopamine neuronal damage in the dense midbrain substantia nigra and dopamine deficiency in the striatum^61^. The substantia nigra contains the largest network of dopaminergic cells in the brain and is involved in the regulation of motor, emotional and cognitive behavior^62^. It was found that rTMS over the M1 region induced endogenous dopamine release in the ventral striatum, which may be its intrinsic mechanism for the treatment of PD^63^. In addition, HF-rTMS over the DLPFC demonstrated favorable improvement in cognition and depression. DLPFC is a core brain region of the central executive network, which is closely related to executive function, attention, and visuospatial ability. It was shown that mood changes in PD patients may be closely related to decreased activity in the left DLPFC. There is still a need for in-depth research on the mechanism of action of NIBS to improve PD, to reveal the scientific basis of its efficacy from neurophysiological and biochemical perspectives, and to conduct large-scale comparative efficacy studies on different targets.

Potential limitations of this study are: (1) inconsistencies in patient age, duration of illness, and severity among the studies included in the analysis may have increased study heterogeneity and affected the results of the analysis; (2) most of the included studies did not explicitly report or implement allocation concealment processes, and more than half of the studies did not implement evaluator blinding; (3) due to language limitations, the literature included in the present study covered only the English and Chinese literature, there is a possibility of incomplete search.

## Conclusions

In summary, HF-rTMS over the SMA may be the best option for improving motor symptoms in PD patients. a-tDCS and HF-rTMS over the M1 may be the NIBS with the most significant effects on cognition and depression, separately. A large number of future RCTs are needed to investigate the efficacy of NIBS in patients with Parkinson's disease and the optimal combination of appropriate parameters, including stimulation frequency and stimulation target.

### Supplementary Information

Supplementary Information.

## Data Availability

Data is provided within the manuscript or supplementary information files.

## Abbreviations

NIBS: Noninvasive brain stimulation
PD: Parkinson’s disease
rTMS: Repetitive transcranial magnetic stimulation
iTBS: Intermittent theta-burst stimulation
tDCS: Transcranial direct current stimulation
UPDRS-III: The motor section of the Unified Parkinson's Disease Rating Scale
MDS-UPDRS-III: The motor section of the Movement Disorder Society Unified Parkinson's Disease Rating Scale
MMSE: Mini-Mental State Examination
MoCA: Montreal Cognitive Assessment
BDI: Beck Depression Inventory
HDRS: Hamilton Depression Rating Scale
