# Supplementary material for: Assessment of noninvasive brain stimulation interventions in Parkinson’s disease: a systematic review and network meta-analysis
Source: Sci Rep. 2024 Jun 20;14:14219. doi: 10.1038/s41598-024-64196-0 (PMC11189909; doi:10.1038/s41598-024-64196-0)
Supplement: Supplementary file 1 — Supplementary Information. [file 41598_2024_64196_MOESM1_ESM.docx]

**Supplementary Table S1:** Search strategies

| **Data source** | **Search terms** | **Total** |
| --- | --- | --- |
| PubMed | (Parkinson OR Parkinsonism OR Parkinson Disease OR Parkinson’ s Disease) AND (noninvasive brain stimulation OR transcranial electrical stimulation OR transcranial direct current stimulation OR transcranial alternating current stimulation OR transcranial random noise stimulation OR transcranial magnetic stimulation OR repetitive transcranial magnetic stimulation OR theta burst stimulation OR intermittent theta burst stimulation OR continuous theta burst stimulation OR NIBS OR TES OR tDCS OR tACS OR tRNS OR TMS OR rTMS OR TBS OR iTBS OR cTBS) AND (randomized controlled trial OR random OR controlled trials OR RCT) | 374 |
| Web of Science | ((TS=((Parkinson OR Parkinsonism OR Parkinson Disease OR Parkinson’ s Disease) )) AND TS=((noninvasive brain stimulation OR transcranial electrical stimulation OR transcranial direct current stimulation OR transcranial alternating current stimulation OR transcranial random noise stimulation OR transcranial magnetic stimulation OR repetitive transcranial magnetic stimulation OR theta burst stimulation OR intermittent theta burst stimulation OR continuous theta burst stimulation OR NIBS OR TES OR tDCS OR tACS OR tRNS OR TMS OR rTMS OR TBS OR iTBS OR cTBS))) AND TS=((randomized controlled trial OR random OR controlled trials OR RCT)) | 404 |
| Cochrane Library | (Parkinson OR Parkinsonism OR Parkinson Disease OR Parkinson’ s Disease) AND (noninvasive brain stimulation OR transcranial electrical stimulation OR transcranial direct current stimulation OR transcranial alternating current stimulation OR transcranial random noise stimulation OR transcranial magnetic stimulation OR repetitive transcranial magnetic stimulation OR theta burst stimulation OR intermittent theta burst stimulation OR continuous theta burst stimulation OR NIBS OR TES OR tDCS OR tACS OR tRNS OR TMS OR rTMS OR TBS OR iTBS OR cTBS) AND (randomized controlled trial OR RCT) | 390 |
| Embase | #1: 'parkinson':ab,ti OR 'parkinsonism':ab,ti OR 'parkinson disease':ab,ti  #2: (((((((((noninvasive AND brain AND stimulation OR transcranial) AND electrical AND stimulation OR transcranial) AND direct AND current AND stimulation OR transcranial) AND alternating AND current AND stimulation OR transcranial) AND random AND noise AND stimulation OR transcranial) AND magnetic AND stimulation OR repetitive) AND transcranial AND magnetic AND stimulation OR theta) AND burst AND stimulation OR intermittent) AND theta AND burst AND stimulation OR continuous) AND theta AND burst AND stimulation OR nibs OR tes OR tdcs OR tacs OR trns OR tms OR rtms OR tbs OR itbs OR ctbs  #3: randomized controlled trial OR RCT  #4: #1 AND #2 AND#3 | 360 |
| CNKI | 经颅磁刺激 + 经颅电刺激 + 经颅直流电刺激 + 重复经颅磁刺激 + 经颅交流电刺激 + 经颅随机噪声刺激（主题） AND 帕金森 + 帕金森病 + 帕金森氏 + 帕金森综合征 + 帕金森病患者 + 帕金森氏病（主题）  Transcranial Magnetic Stimulation + Transcranial Electrical Stimulation + Transcranial DC Stimulation + Repetitive Transcranial Magnetic Stimulation + Transcranial AC Stimulation + Transcranial Random Noise Stimulation (Subject) AND Parkinson's + Parkinson's Disease + Parkinson's Syndrome + People with Parkinson's Disease (Subject) | 316 |
| Wanfang database | 经颅磁刺激 OR 经颅电刺激 OR 经颅直流电刺激 OR 重复经颅磁刺激 OR 经颅交流电刺激 OR 经颅随机噪声刺激（题名或关键词） AND 帕金森 OR 帕金森病 OR 帕金森氏 OR 帕金森综合征 OR 帕金森病患者 OR 帕金森氏病（题名或关键词）  Transcranial magnetic stimulation OR Transcranial electrical stimulation OR Transcranial direct current stimulation OR Repetitive transcranial magnetic stimulation OR Transcranial alternating current stimulation OR Transcranial random noise stimulation (title or keyword) AND Parkinson's OR Parkinson's Disease OR Parkinson's Syndrome OR People with Parkinson's Disease (title or keyword) | 439 |
| VIP | 经颅磁刺激 OR 经颅电刺激 OR 经颅直流电刺激 OR 重复经颅磁刺激 OR 经颅交流电刺激 OR 经颅随机噪声刺激（题名或关键词） AND 帕金森 OR 帕金森病 OR 帕金森氏 OR 帕金森综合征 OR 帕金森病患者 OR 帕金森氏病（题名或关键词）  Transcranial magnetic stimulation OR Transcranial electrical stimulation OR Transcranial direct current stimulation OR Repetitive transcranial magnetic stimulation OR Transcranial alternating current stimulation OR Transcranial random noise stimulation (title or keyword) AND Parkinson's OR Parkinson's Disease OR Parkinson's Syndrome OR People with Parkinson's Disease (title or keyword) | 361 |
| SinoMed | ( "经颅磁刺激"[常用字段:智能] OR "经颅电刺激"[常用字段:智能] OR "经颅直流电刺激"[常用字段:智能] OR "重复经颅磁刺激"[常用字段:智能] OR "经颅交流电刺激"[常用字段:智能] OR "经颅随机噪声刺激"[常用字段:智能]) AND "帕金森"[常用字段:智能]  ("transcranial magnetic stimulation"[common field:smart] OR "transcranial electrical stimulation"[common field:smart] OR "transcranial direct current stimulation"[common field:smart] OR "repetitive transcranial magnetic stimulation"[common field:smart] OR "transcranial alternating current stimulation"[common field:smart] OR "transcranial random noise stimulation"[common field:smart]) AND "Parkinson"[common field :smart] | 392 |
| ClinicalTrials.gov | (non-invasive brain stimulation) AND (Parkinson OR Parkinsonism OR Parkinson Disease OR Parkinson’ s Disease) | 15 |
| Total | up to April 30th, 2024 | 3051 |

**Supplementary Table S2:** Excluded studies and reason.

| **Reason** | **Numbers** | **References** |
| --- | --- | --- |
| Intervention modality not NIBS stimulation and sham stimulation | 8 | 1-8 |
| Intervention not only NIBS stimulation | 2 | 9,10 |
| Treatment duration too short (<5 days) | 2 | 11,12 |
| Irrelevant outcomes | 21 | 13-33 |
| Study data not available | 4 | 34-37 |
| Not RCT | 18 | 38-55 |
| Meta-analysis | 10 | 56-65 |
| Review article | 6 | 66-71 |
| Protocol but not report of study result | 5 | 72-76 |

**References**

1 Morberg, B. M. *et al.* Effects of transcranial pulsed electromagnetic field stimulation on quality of life in Parkinson's disease. *Eur J Neurol* **25**, 963-e974 (2018). <https://doi.org:10.1111/ene.13637>

2 Zhou, Y. *et al.* The Effect of Propofol versus Sevoflurane on Postoperative Delirium in Parkinson’s Disease Patients Undergoing Deep Brain Stimulation Surgery: an Observational Study. *Brain sciences* **12** (2022). <https://doi.org:10.3390/brainsci12060689>

3 Wilhelm, E., Quoilin, C., Derosiere, G., Jeanjean, A. & Duque, J. The role of Dopamine in Preparatory Inhibition: what can we learn from Parkinson's disease? *Movement disorder* **34**, S465 (2019).

4 Urwyler, P. *et al.* INFLUENCE OF PRE-STIMULATION RHYTHMS ON PHOSPHENE PERCEPTION IN THE CONTEXT OF VISUAL HALLUCINATIONS. *Alzheimer's and Dementia* **14**, P1557-P1558 (2018). <https://doi.org:10.1016/j.jalz.2018.07.107>

5 Strafella, A. P. *et al.* Effects of chronic levodopa and pergolide treatment on cortical excitability in patients with Parkinson's disease: a transcranial magnetic stimulation study. *Clin Neurophysiol* **111**, 1198-1202 (2000). <https://doi.org:10.1016/s1388-2457(00)00316-3>

6 Chen, J., Xu, P., Guo, X. & Zou, T. Comparative Analysis of the Effects of Escitalopram, Pramipexole, and Transcranial Magnetic Stimulation on Depression in Patients With Parkinson Disease: An Open-Label Randomized Controlled Trial. *Clin Neuropharmacol* **45**, 84-88 (2022). <https://doi.org:10.1097/wnf.0000000000000507>

7 Costa-Ribeiro, A. *et al.* Transcranial direct current stimulation associated with gait training in Parkinson's disease: A pilot randomized clinical trial. *Dev Neurorehabil* **20**, 121-128 (2017). <https://doi.org:10.3109/17518423.2015.1131755>

8 Kwon, D. Y., Park, M. H., Yoon, H. K. & Kim, J. H. Clinical effects of anodal transcranial direct current stimulation on the amount of daily activity and sleep in Parkinson's disease patients. *Clinical neurophysiology* **131**, e153 (2020). <https://doi.org:10.1016/j.clinph.2019.12.350>

9 Dashtelei, A. A. *et al.* Adjunctive transcranial direct current stimulation to improve swallowing functions in Parkinson's disease. *Excli j* **23**, 95-107 (2024). <https://doi.org:10.17179/excli2023-6496>

10 Pastore-Wapp, M. *et al.* Feasibility of a combined intermittent theta-burst stimulation and video game-based dexterity training in Parkinson's disease. *J Neuroeng Rehabil* **20**, 2 (2023). <https://doi.org:10.1186/s12984-023-01123-w>

11 Maruo, T. *et al.* High-frequency repetitive transcranial magnetic stimulation over the primary foot motor area in Parkinson's disease. *Brain Stimul* **6**, 884-891 (2013). <https://doi.org:10.1016/j.brs.2013.05.002>

12 Yokoe, M. *et al.* The optimal stimulation site for high-frequency repetitive transcranial magnetic stimulation in Parkinson's disease: A double-blind crossover pilot study. *J Clin Neurosci* **47**, 72-78 (2018). <https://doi.org:10.1016/j.jocn.2017.09.023>

13 Yadolahi, F. & Hosseini, N. TU-167. Cumulative effects of combined rTMS and Perturbation Treadmill Training in Gait Performance in Parkinson Disease: a Pilot Study. *Clinical neurophysiology* **141**, S27 (2022). <https://doi.org:10.1016/j.clinph.2022.07.071>

14 Workman, C. D., Fietsam, A. C., Uc, E. Y. & Rudroff, T. Cerebellar transcranial direct current stimulation in people with parkinson’s disease: a pilot study. *Brain sciences* **10** (2020). <https://doi.org:10.3390/brainsci10020096>

15 Schabrun, S. M., Lamont, R. M. & Brauer, S. G. Transcranial Direct Current Stimulation to Enhance Dual-Task Gait Training in Parkinson's Disease: A Pilot RCT. *PLoS One* **11**, e0158497 (2016). <https://doi.org:10.1371/journal.pone.0158497>

16 Schabrun, S., Lamont, R. & Brauer, S. A pilot, randomized, double-blind, sham-controlled trial of transcranial direct current stimulation to enhance dual-task gait training in people with Parkinson's disease. *Journal of Parkinson's disease* **6**, 203‐204 (2016). <https://doi.org:10.3233/JPD-169900>

17 Nojima, I. *et al.* Gait-combined closed-loop brain stimulation can improve walking dynamics in Parkinsonian gait disturbances: a randomised-control trial. *J Neurol Neurosurg Psychiatry* (2023). <https://doi.org:10.1136/jnnp-2022-329966>

18 Makkos-Weisz, A., Kovacs, N., Endre, P., Kovacs, M. & Pinter, D. Repetitive transcranial magnetic stimulation can improve anxiety in Parkinson's disease: a randomized, double-blind and controlled trial. *Movement disorder* **34**, S393‐S394 (2019).

19 Lima Silva, D. C. D. *et al.* Effects of Acute Transcranial Direct Current Stimulation on Gait Kinematics of Individuals With Parkinson Disease. *Topics in geriatric rehabilitation* **34**, 262‐268 (2018). <https://doi.org:10.1097/TGR.0000000000000203>

20 Kovacs, N., Pal, E., Weisz, A. M., Kovacs, M. & Pinter, D. Repetitive transcranial magnetic stimulation can improve anxiety in Parkinson's disease: a randomized, double-blind and controlled trial. *Brain stimulation* **12**, 409 (2019). <https://doi.org:10.1016/j.brs.2018.12.320>

21 Zhang, X. *et al.* Effects of repetitive transcranial magnetic stimulation over right dorsolateral prefrontal cortex on excessive daytime sleepiness in patients with Parkinson's disease. *Sleep medicine* **100**, 133‐138 (2022). <https://doi.org:10.1016/j.sleep.2022.08.003>

22 Zamorano, Y. G. *et al.* Efectiveness of transcranial Direct Current Stimulation (tDCS) on Parkinson´s Disease (PD)-related pain: effects on pain processing features in PD patients. *Brain stimulation* **16**, 10 (2023). <https://doi.org:10.1016/j.brs.2023.03.039>

23 Yotnuengnit, P., Bhidayasiri, R., Donkhan, R., Chaluaysrimuang, J. & Piravej, K. Effects of Transcranial Direct Current Stimulation Plus Physical Therapy on Gait in Patients With Parkinson Disease: A Randomized Controlled Trial. *Am J Phys Med Rehabil* **97**, 7-15 (2018). <https://doi.org:10.1097/phm.0000000000000783>

24 Yang, Y. R. *et al.* Combination of rTMS and treadmill training modulates corticomotor inhibition and improves walking in Parkinson disease: a randomized trial. *Neurorehabil Neural Repair* **27**, 79-86 (2013). <https://doi.org:10.1177/1545968312451915>

25 Mak, M. K. Repetitive transcranial magnetic stimulation combined with treadmill training can modulate corticomotor inhibition and improve walking performance in people with Parkinson's disease. *J Physiother* **59**, 128 (2013). <https://doi.org:10.1016/s1836-9553(13)70167-x>

26 Aksu, S. *et al.* Does transcranial direct current stimulation enhance cognitive performance in Parkinson's disease mild cognitive impairment? An event-related potentials and neuropsychological assessment study. *Neurol Sci* **43**, 4029-4044 (2022). <https://doi.org:10.1007/s10072-022-06020-z>

27 Wong, P. L. *et al.* Transcranial Direct Current Stimulation on Different Targets to Modulate Cortical Activity and Dual-Task Walking in Individuals With Parkinson's Disease: A Double Blinded Randomized Controlled Trial. *Front Aging Neurosci* **14**, 807151 (2022). <https://doi.org:10.3389/fnagi.2022.807151>

28 Wei, W. *et al.* Acute improvement in the attention network with repetitive transcranial magnetic stimulation in Parkinson's disease. *Disabil Rehabil* **44**, 7958-7966 (2022). <https://doi.org:10.1080/09638288.2021.2004245>

29 Wang, H. *et al.* Increased cerebellar activation after repetitive transcranial magnetic stimulation over the primary motor cortex in patients with multiple system atrophy. *Ann Transl Med* **4**, 103 (2016). <https://doi.org:10.21037/atm.2016.03.24>

30 González-Zamorano, Y. *et al.* TDCS for parkinson's disease disease-related pain: A randomized trial. *Clin Neurophysiol* **161**, 133-146 (2024). <https://doi.org:10.1016/j.clinph.2024.01.011>

31 Murdoch, B. E., Ng, M. L. & Barwood, C. H. Treatment of articulatory dysfunction in Parkinson's disease using repetitive transcranial magnetic stimulation. *Eur J Neurol* **19**, 340-347 (2012). <https://doi.org:10.1111/j.1468-1331.2011.03524.x>

32 Lima de Albuquerque, L. *et al.* An Acute Application of Cerebellar Transcranial Direct Current Stimulation Does Not Improve Motor Performance in Parkinson's Disease. *Brain Sci* **10** (2020). <https://doi.org:10.3390/brainsci10100735>

33 Lang, S. T. *et al.* Theta-burst Stimulation for Cognitive Enhancement in Parkinson's Disease With Mild Cognitive Impairment: A Randomized, Double-Blind, Sham-Controlled Trial. *Clinical Neurosurgery* **67**, 214 (2020). <https://doi.org:10.1093/neuros/nyaa447-625>

34 Makkos, A. *et al.* High-Frequency Repetitive Transcranial Magnetic Stimulation Can Improve Depression in Parkinson's Disease: A Randomized, Double-Blind, Placebo-Controlled Study. *Neuropsychobiology* **73**, 169-177 (2016). <https://doi.org:10.1159/000445296>

35 Pal, E., Nagy, F., Aschermann, Z., Balazs, E. & Kovacs, N. The impact of left prefrontal repetitive transcranial magnetic stimulation on depression in Parkinson's disease: a randomized, double-blind, placebo-controlled study. *Mov Disord* **25**, 2311-2317 (2010). <https://doi.org:10.1002/mds.23270>

36 Cohen, O. S. *et al.* Repetitive Deep TMS for Parkinson Disease: A 3-Month Double-Blind, Randomized Sham-Controlled Study. *J Clin Neurophysiol* **35**, 159-165 (2018). <https://doi.org:10.1097/wnp.0000000000000455>

37 Doruk, D., Gray, Z., Bravo, G. L., Pascual-Leone, A. & Fregni, F. Effects of tDCS on executive function in Parkinson's disease. *Neurosci Lett* **582**, 27-31 (2014). <https://doi.org:10.1016/j.neulet.2014.08.043>

38 Mishra, R. K. & Thrasher, A. T. Transcranial direct current stimulation of dorsolateral prefrontal cortex improves dual-task gait performance in patients with Parkinson's disease: a double blind, sham-controlled study. *Gait & posture* **84**, 11‐16 (2021). <https://doi.org:10.1016/j.gaitpost.2020.11.012>

39 Zhang, B., Huang, F., Liu, J. & Zhang, D. Bilateral transcranial direct current stimulation may be a feasible treatment of Parkinsonian tremor. *Front Neurosci* **17**, 1101751 (2023). <https://doi.org:10.3389/fnins.2023.1101751>

40 Torres, J. A. K., Abou Zaki, S. D. & Rosales, R. Clinical motor outcomes immediately after repetitive transcranial magnetic stimulation (rTMS) for Parkinson Disease in a Filipino cohort. *Movement Disorders Clinical Practice* **6**, S172 (2019). <https://doi.org:10.1002/mdc3.12758>

41 Lee, S. Y. *et al.* Effects of repetitive transcranial magnetic stimulation on freezing of gait in patients with Parkinsonism. *Restor Neurol Neurosci* **32**, 743-753 (2014). <https://doi.org:10.3233/rnn-140397>

42 Zhuang, S. *et al.* Improvement of excessive daytime sleepiness with low frequency repetitive transcranial magnetic stimulation in Parkinson's disease: a preliminary Sham-Controlled study. *Movement disorder* **36**, S447‐S448 (2021). <https://doi.org:10.1002/mds.28794>

43 Van Der Werf, Y. D., Sadikot, A. F., Strafella, A. P. & Paus, T. The neural response to transcranial magnetic stimulation of the human motor cortex. II. Thalamocortical contributions. *EXPERIMENTAL BRAIN RESEARCH* **175**, 246-255 (2006). <https://doi.org:10.1007/s00221-006-0548-x>

44 Udupa, K. *et al.* Cortical Plasticity Induction by Pairing Subthalamic Nucleus Deep-Brain Stimulation and Primary Motor Cortical Transcranial Magnetic Stimulation in Parkinson's Disease. *J Neurosci* **36**, 396-404 (2016). <https://doi.org:10.1523/jneurosci.2499-15.2016>

45 Tard, C., Devanne, H., Defebvre, L. & Delval, A. Single session intermittent theta-burst stimulation on the left premotor cortex does not alleviate freezing of gait in Parkinson's disease. *Neurosci Lett* **628**, 1-9 (2016). <https://doi.org:10.1016/j.neulet.2016.05.061>

46 Swank, C., Mehta, J. & Criminger, C. Transcranial direct current stimulation lessens dual task cost in people with Parkinson's disease. *Neurosci Lett* **626**, 1-5 (2016). <https://doi.org:10.1016/j.neulet.2016.05.010>

47 Simonetta, C. *et al.* Motor cortex transcranial direct current stimulation improves non-motor symptoms in early-onset Parkinson's disease: a pilot study. *J Neural Transm (Vienna)* **131**, 189-193 (2024). <https://doi.org:10.1007/s00702-023-02726-2>

48 Srovnalova, H., Marecek, R., Kubikova, R. & Rektorova, I. The role of the right dorsolateral prefrontal cortex in the Tower of London task performance: repetitive transcranial magnetic stimulation study in patients with Parkinson's disease. *Exp Brain Res* **223**, 251-257 (2012). <https://doi.org:10.1007/s00221-012-3255-9>

49 Rashid-Lopez, R. *et al.* Marked improvement of postural and gait disturbances in Parkinson's disease with bilateral primary motor area intermittent theta-burst stimulation may be linked to increased putamen-cortico-cerebellar functional connectivity: a case report. *Brain stimulation* **16**, 271‐272 (2023). <https://doi.org:10.1016/j.brs.2023.01.459>

50 Minks, E., Marecek, R., Pavlik, T., Chroust, K. & Bares, M. The effect of repetitive transcranial magnetic stimulation of the cerebellum on the upper limb performance in early parkinson's disease - Pilot study. *Ceska a slovenska neurologie a neurochirurgie* **73**, 32‐36 (2010).

51 Lima De Albuquerque, L., Fischer, K., Jalene, S., Landers, M. R. & Poston, B. The influence of cerebellar transcranial direct current stimulation on skill acquisition in Parkinson's disease. *Movement Disorders* **31**, S658-S659 (2016). <https://doi.org:10.1002/mds.26688>

52 Lau, C. I. *et al.* Effect of single-session transcranial direct current stimulation on cognition in Parkinson's disease. *CNS Neurosci Ther* **25**, 1237-1243 (2019). <https://doi.org:10.1111/cns.13210>

53 Capecci, M., Andrenelli, E., Orni, C. & Ceravolo, M. G. Bilateral prefrontal transcranial direct current stimulation (tDCS) in Parkinson's disease: a placebo controlled trial. *Movement disorders* **29**, S229‐S230 (2014). <https://doi.org:10.1002/mds.25914>

54 Filipović, S. R., Rothwell, J. C. & Bhatia, K. Low-frequency repetitive transcranial magnetic stimulation and off-phase motor symptoms in Parkinson's disease. *J Neurol Sci* **291**, 1-4 (2010). <https://doi.org:10.1016/j.jns.2010.01.017>

55 Bonello, M., Nurmikko, T., Mavrianou, A. & Steiger, M. Repetitive transcranial magnetic stimulation (rTMS) for the treatment of pain in Parkinson's Disease-an open-label study. *Movement disorder* **34**, S624‐S626 (2019).

56 Zheng, H. B. *et al.* Non-invasive brain stimulation for treating psychiatric symptoms in Parkinson's disease: A systematic review and meta-analysis. *J Clin Neurosci* **106**, 83-90 (2022). <https://doi.org:10.1016/j.jocn.2022.10.013>

57 Lawrence, B. J., Gasson, N., Bucks, R. S., Troeung, L. & Loftus, A. M. Cognitive Training and Noninvasive Brain Stimulation for Cognition in Parkinson's Disease: A Meta-analysis. *Neurorehabil Neural Repair* **31**, 597-608 (2017). <https://doi.org:10.1177/1545968317712468>

58 Kim, Y. W., Shin, I. S., Moon, H. I., Lee, S. C. & Yoon, S. Y. Effects of non-invasive brain stimulation on freezing of gait in parkinsonism: A systematic review with meta-analysis. *Parkinsonism Relat Disord* **64**, 82-89 (2019). <https://doi.org:10.1016/j.parkreldis.2019.02.029>

59 Zhu, H. *et al.* Low-frequency repetitive transcranial magnetic stimulation on Parkinson motor function: a meta-analysis of randomised controlled trials. *Acta Neuropsychiatr* **27**, 82-89 (2015). <https://doi.org:10.1017/neu.2014.43>

60 Zhou, L. *et al.* Antidepressant Effects of Repetitive Transcranial Magnetic Stimulation Over Prefrontal Cortex of Parkinson's Disease Patients With Depression: A Meta-Analysis. *Front Psychiatry* **9**, 769 (2018). <https://doi.org:10.3389/fpsyt.2018.00769>

61 Xie, Y. J., Gao, Q., He, C. Q. & Bian, R. Effect of Repetitive Transcranial Magnetic Stimulation on Gait and Freezing of Gait in Parkinson Disease: A Systematic Review and Meta-analysis. *Arch Phys Med Rehabil* **101**, 130-140 (2020). <https://doi.org:10.1016/j.apmr.2019.07.013>

62 Wang, P., Gou, Y., Liao, W. J. & Li, H. G. Effectiveness of high- and low-frequency repetitive transcranial magnetic stimulation for treating dysfunction in patients with Parkinson's disease: A meta-analysis. *Chinese Journal of Evidence-Based Medicine* **10**, 1308-1315 (2010).

63 Wagle Shukla, A. *et al.* Repetitive Transcranial Magnetic Stimulation (rTMS) Therapy in Parkinson Disease: A Meta-Analysis. *PM and R* **8**, 356-366 (2016). <https://doi.org:10.1016/j.pmrj.2015.08.009>

64 Tsai, P.-Y., Chen, Y.-C., Wang, J.-Y., Chung, K.-H. & Lai, C.-H. Effect of repetitive transcranial magnetic stimulation on depression and cognition in individuals with traumatic brain injury: a systematic review and meta-analysis. *SCIENTIFIC REPORTS* **11** (2021). <https://doi.org:10.1038/s41598-021-95838-2>

65 Nguyen, T. X. D., Mai, P. T., Chang, Y. J. & Hsieh, T. H. Effects of transcranial direct current stimulation alone and in combination with rehabilitation therapies on gait and balance among individuals with Parkinson's disease: a systematic review and meta-analysis. *J Neuroeng Rehabil* **21**, 27 (2024). <https://doi.org:10.1186/s12984-024-01311-2>

66 Madrid, J. & Benninger, D. H. Non-invasive brain stimulation for Parkinson's disease: Clinical evidence, latest concepts and future goals: A systematic review. *J Neurosci Methods* **347**, 108957 (2021). <https://doi.org:10.1016/j.jneumeth.2020.108957>

67 Wu, Y. *et al.* Transcranial Magnetic Stimulation Alleviates Levodopa-Induced Dyskinesia in Parkinson's Disease and the Related Mechanisms: A Mini-Review. *Front Neurol* **12**, 758345 (2021). <https://doi.org:10.3389/fneur.2021.758345>

68 Tsuji, S. & Akamatsu, N. Does transcranial magnetic stimulation improve the motor symptoms of Parkinson disease? *J Neurol* **250 Suppl 3**, Iii47-50 (2003). <https://doi.org:10.1007/s00415-003-1309-4>

69 Rektorová, I. & Anderková, Ľ. Noninvasive Brain Stimulation and Implications for Nonmotor Symptoms in Parkinson's Disease. *Int Rev Neurobiol* **134**, 1091-1110 (2017). <https://doi.org:10.1016/bs.irn.2017.05.009>

70 Pol, F., Salehinejad, M. A., Baharlouei, H. & Nitsche, M. A. The effects of transcranial direct current stimulation on gait in patients with Parkinson's disease: a systematic review. *Transl Neurodegener* **10**, 22 (2021). <https://doi.org:10.1186/s40035-021-00245-2>

71 Ni, R. *et al.* Novel Non-invasive Transcranial Electrical Stimulation for Parkinson’s Disease. *Frontiers in Aging Neuroscience* **14** (2022). <https://doi.org:10.3389/fnagi.2022.880897>

72 Sharma, K. *et al.* Remotely supervised transcranial direct current stimulation (RSTDCS) to mitigate fatigue and cognitive decline: a novel protocol for Parkinson's disease. *Movement disorders* **33**, S48‐S49 (2018). <https://doi.org:10.1002/mds.27434>

73 Nascimento, L. R. *et al.* Transcranial direct current stimulation (tDCS) in addition to walking training on walking, mobility, and reduction of falls in Parkinson's disease: study protocol for a randomized clinical trial. *Trials* **22**, 647 (2021). <https://doi.org:10.1186/s13063-021-05603-z>

74 Li, P. *et al.* Neuroprotective Effects of Intermittent Theta Burst Stimulation in Parkinson's Disease (NET-PD): A Study Protocol for a Delayed-Start Randomized Double-Blind Sham-Controlled Trial. *J Clin Med* **11** (2022). <https://doi.org:10.3390/jcm11174972>

75 Jin, Z. H. *et al.* Intermittent theta-burst stimulation combined with physical therapy as an optimal rehabilitation in Parkinson's disease: study protocol for a randomised, double-blind, controlled trial. *Trials* **24**, 410 (2023). <https://doi.org:10.1186/s13063-023-07425-7>

76 Alizad, V. *et al.* Effects of transcranial direct current stimulation on gait in people with Parkinson's disease: study protocol for a randomized, controlled clinical trial. *Trials* **19**, 661 (2018). <https://doi.org:10.1186/s13063-018-2982-z>

**Supplementary Table S3:** Inconsistency test: motor function.

| **Side** | **Direct** | | **Indirect** | | **Difference** | | | **Treatments used** |
| --- | --- | --- | --- | --- | --- | --- | --- | --- |
|  | **Coef.** | **Std. Err.** | **Coef.** | **Std. Err.** | **Coef.** | **Std. Err.** | **P>z** |  |
| A B | . | . | . | . | . | . | . | A: Sham |
| A C | -0.5262294 | 0.5408122 | -1.391505 | 0.5886312 | 0.8652759 | 0.7993526 | 0.279 | B: HF-rTMS-M1+DLPFC |
| A D | -0.6990539 | 0.3002508 | -0.7198573 | 0.647621 | 0.0208034 | 0.7138357 | 0.977 | C: HF-rTMS-M1+PFC |
| A E | -0.9902069 | 0.3203088 | -1.236554 | 0.3499337 | 0.2463473 | 0.474405 | 0.604 | D: HF-rTMS-DLPFC |
| A F | . | . | . | . | . | . | . | E: HF-rTMS-M1 |
| A G | -0.3212427 | 0.3727967 | -0.3004366 | 0.6087581 | -0.020806 | 0.7138359 | 0.977 | F: HF-rTMS-SMA |
| A H | -0.915689 | 0.2792474 | -0.3510339 | 0.3834359 | -0.5646551 | 0.4744795 | 0.234 | G: LF-rTMS-DLPFC |
| A I | . | . | . | . | . | . | . | H: LF-rTMS-M1 |
| A J | . | . | . | . | . | . | . | I: iTBS-M1+DLPFC |
| A K | . | . | . | . | . | . | . | J: a-tDCS-M1+SMA |
| A L | . | . | . | . | . | . | . | K: a-tDCS-DLPFC |
| C E | 0.2101744 | 0.5377688 | -0.655105 | 0.5914132 | 0.8652795 | 0.7993528 | 0.279 | L: a-tDCS-M1 |
| D G | 0.3986161 | 0.5295627 | 0.3778114 | 0.4786712 | 0.0208047 | 0.7138367 | 0.977 |  |
| E H | 0.5460196 | 0.254293 | -0.0186327 | 0.4008673 | 0.5646523 | 0.4744801 | 0.234 |  |

**Supplementary Table S4:** Inconsistency test: cognitive function.

| **Side** | **Direct** | | **Indirect** | | **Difference** | | | **Treatments used** |
| --- | --- | --- | --- | --- | --- | --- | --- | --- |
|  | **Coef.** | **Std. Err.** | **Coef.** | **Std. Err.** | **Coef.** | **Std. Err.** | **P>z** |  |
| A B | . | . | . | . | . | . | . | A: Sham |
| A C | 0.1358512 | 0.7005574 | 1.352476 | 0.8247814 | -1.216625 | 1.082148 | 0.261 | B: HF-rTMS-M1+DLPFC |
| A D | . | . | . | . | . | . | . | C: HF-rTMS-M1+PFC |
| A E | 0.7614301 | 0.5182445 | 0.7912373 | 0.7278648 | -0.0298072 | 0.8932656 | 0.973 | D: HF-rTMS-DLPFC |
| A F | 1.089154 | 0.6732569 | -0.0878122 | 0.8007428 | 1.176966 | 1.046166 | 0.261 | E: HF-rTMS-M1 |
| A G | . | . | . | . | . | . | . | F: LF-rTMS-M1 |
| A H | . | . | . | . | . | . | . | G: iTBS-DLPFC |
| A I | . | . | . | . | . | . | . | H: a-tDCS-DLPFC |
| A J | . | . | . | . | . | . | . | I: a-tDCS-M1 |
| C E | -0.3823192 | 0.7018947 | 0.834889 | 0.8237444 | -1.217208 | 1.082225 | 0.261 | J: c-tDCS-DLPFC |
| E F | -0.6554059 | 0.6693932 | 0.5213529 | 0.8040825 | -1.176759 | 1.046248 | 0.261 |  |

**Supplementary Table S5:** Inconsistency test: depression.

| **Side** | **Direct** | | **Indirect** | | **Difference** | | | **Treatments used** |
| --- | --- | --- | --- | --- | --- | --- | --- | --- |
|  | **Coef.** | **Std. Err.** | **Coef.** | **Std. Err.** | **Coef.** | **Std. Err.** | **P>z** |  |
| A B | . | . | . | . | . | . | . | A: Sham |
| A C | 0.0345307 | 0.7051805 | -1.691395 | 0.8244691 | 1.725926 | 1.08491 | 0.112 | B: HF-rTMS-M1+DLPFC |
| A D | -0.7962227 | 0.3807879 | -0.7019429 | 1.085577 | -0.0942798 | 1.150425 | 0.935 | C: HF-rTMS-M1+PFC |
| A E | -1.323713 | 0.5535095 | -1.61595 | 0.7134191 | 0.2922362 | 0.9029846 | 0.746 | D: HF-rTMS-DLPFC |
| A F | -0.0898302 | 0.7628993 | -0.0957832 | 1.078581 | 0.005953 | 1.321118 | 0.996 | E: HF-rTMS-M1 |
| A G | -0.2926189 | 0.7676447 | -0.3861993 | 0.8572339 | 0.0935803 | 1.150708 | 0.935 | F: HF-rTMS-SMA |
| A H | -1.837706 | 0.653574 | -0.1143421 | 0.6184206 | -1.723364 | 0.8997794 | 0.055 | G: LF-rTMS-DLPFC |
| A I | 0.0516557 | 0.7628723 | 0.057642 | 1.078601 | -0.0059863 | 1.321119 | 0.996 | H: LF-rTMS-M1 |
| A J | . | . | . | . | . | . | . | I: LF-rTMS-SMA |
| C E | -0.013357 | 0.7051734 | -1.740145 | 0.8245745 | 1.726788 | 1.084985 | 0.111 | J: a-tDCS-DLPFC |
| D G | 0.4097633 | 0.7681428 | 0.5037245 | 0.8568804 | -0.0939612 | 1.150777 | 0.935 |  |
| E H | 0.9399678 | 0.4489845 | -0.7827109 | 0.7798447 | 1.722679 | 0.899859 | 0.056 |  |
| F I | 0.1474581 | 0.7629892 | 0.1414859 | 1.078909 | 0.0059723 | 1.321438 | 0.996 |  |


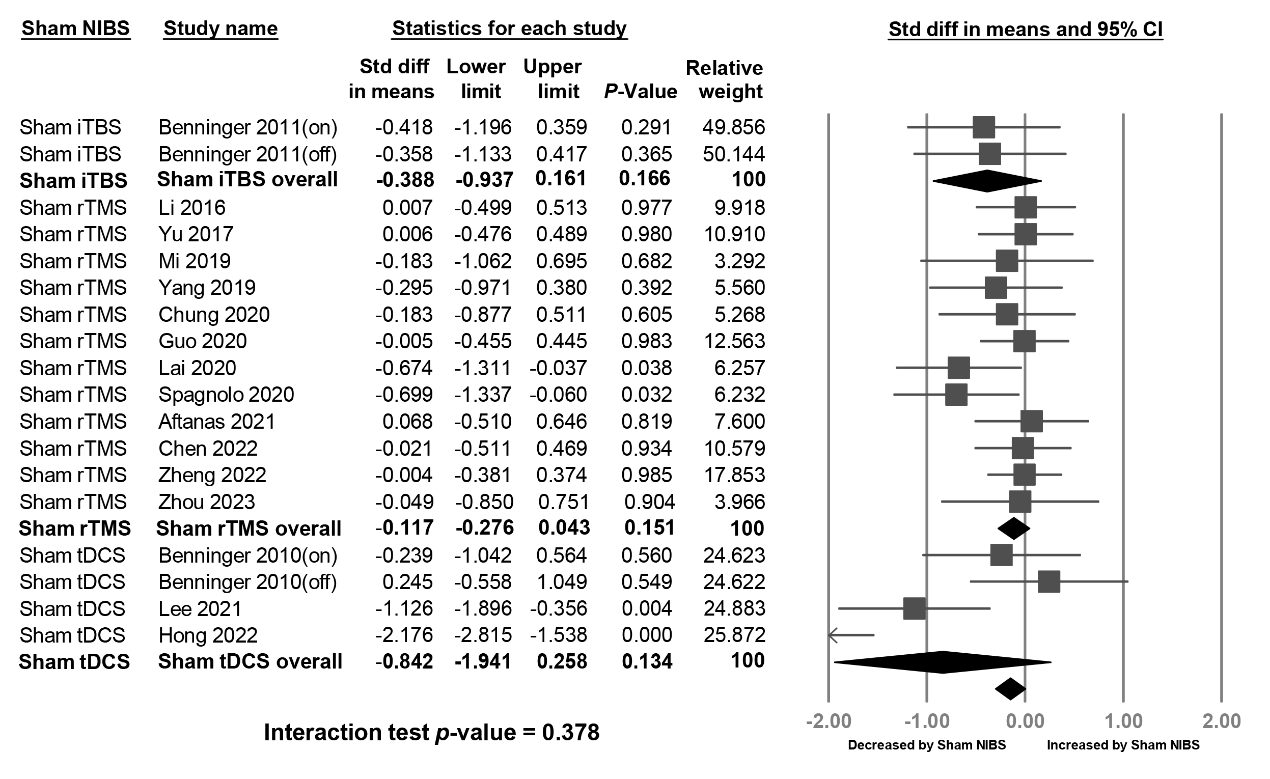
**Supplementary Figure 1.** Test for transitivity assumption of motor function scores.


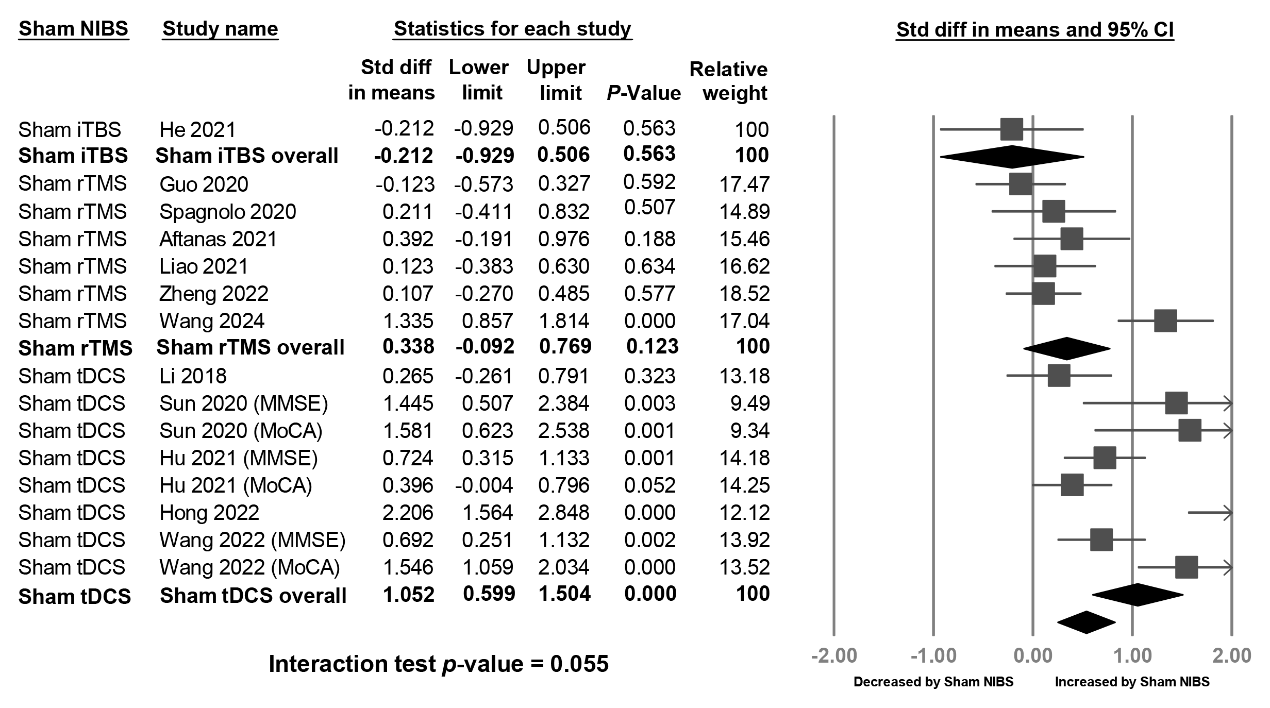
**Supplementary Figure 2.** Test for transitivity assumption of cognitive function scores.


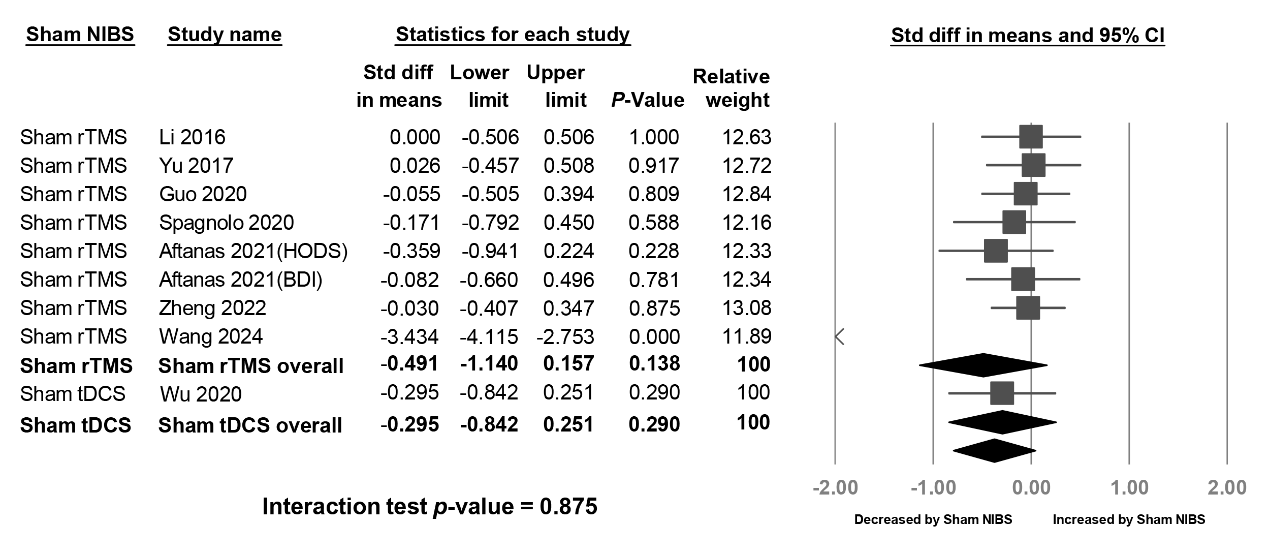
**Supplementary Figure 3.** Test for transitivity assumption of cognitive depression scores.
